# Exposed Pulps,—Secondary Dentine

**Published:** 1860-10

**Authors:** 


### EXPOSED PULPS—SECONDARY DENTINE.

BY GEO. WATT.

There is some diversity of sentiment, among dentists, on the subject of exposed pulps. This is refreshingly evident, from the reports of discussions, at the late meeting of the American Convention. Diversity of opinion, on an important subject which is but partially understood, is an evidence that progress is about to be made toward its thorough investigation. Some points, in regard to this subject, appear to be already settled. All agree that a living, healthy pulp is important to the well-being of the tooth. All admit that exposure of the pulp is a misfortune. Every dentist endeavors to avoid its unnecessary exposure. But when this misfortune has happened—when the pulp is exposed, what is to be done? That is the question.

Many prominent dentists—a majority, perhaps—advocate the extirpation of the pulp, in all cases of exposure. The slightest exposure of the pulp of the most important tooth of the healthiest patient, meets with no mercy at their hands. The pulp is exposed. That is sufficient. It must be destroyed. If the profession is to be restricted to but one mode of practice, in the treatment of exposed pulps, that of extirpation is, doubtless, the one to be adopted. There are some, however, who claim that, in many cases, there is "a more excellent way."

While they treat, by extirpation, a large majority of the cases of exposed pulps presented to them, there are some who maintain that we should discriminate, in regard to the character of the cases referred to us. They believe that a slight exposure of a healthy pulp, in a good constitution, does not call for extirpation. They claim that the pulp, under such circumstances, may be preserved by proper treatment, and that, consequently, the tooth will be much more likely to be permanently useful, than if it were destroyed.

This is a very important question. There is none before the profession more so. In its investigation, all prejudices should be laid aside. No one can afford to be in error here. If some teeth can be saved alive, throughout their entire structure, after their pulps are exposed, it is important to know what ones. If none such can be saved, it is as important to know it ; for those who try to save them, merely torture their patients, and bring reproach on their profession.

Let us, then, inquire what is the exact condition of a pulp that is merely exposed, but not diseased. And by the term pulp, here, without aiming to be technical, we include all the soft tissues found in the interior of a normal tooth.

The pulp is a highly organized soft tissue. It is well supplied with both vessels and nerves ; and the diameter of the former is sufficient to admit the red globules, and, consequently, all the constituents of the blood. It has the ordinary physiological properties and functions of other soft tissues, and is, therefore, liable to all the ordinary pathological changes of such tissues. It may be wounded ; and the wound may heal. It is liable to inflammation ; which may terminate in resolution, suppuration, ulceration, or mortification. Like other soft tissues, it admits of contact with some foreign bodies, without being pained. And, like them, it may tolerate such contact for some time, without taking on inflammation, or even irritation. It is covered by, or invested with the lining membrane of the pulp cavity, and has a vital connection with it. This membrane bears the same relation to dentine that the periosteum does to ordinary bone, and performs corresponding functions. It is the medium of connection between the soft parts of the system and the dentine. It supplies ossific matter to the dentine, as the periosteum does to bone. By the normal fulfillment of its function, the pulp cavity is lessened, as age advances; and, by an increase, or quickening of this function, the pulp is often protected from exposure by the wearing away of the teeth, or by caries. Both the physical and chemical properties of dentine vary with the condition of this membrane while depositing it. When there is nothing to disturb its functions, ordinary dentine is deposited by it. When its vital energies are aroused, "secondary," or rather, modified dentine is formed; and when its vital force is depressed, or there is a lack of ossific matter in the blood, this modified dentine is often porous and irregular, and, sometimes, closely resembles cementum.

Now, it is a well recognized fact that the periosteum will deposit bone, even when deprived of its ordinary connection with bony structures. All that is necessary is for it to have a vital union with the general circulation. When stripped from the bone, and transplanted, it still performs its normal functions. A bone may be lost by necrosis, but, if the periosteum remain, a new bone will be formed, resembling the lost one in shape in proportion as the integrity of the periosteum was preserved.

Bearing these principles in mind, we will endeavor to apply them, shortly.

The propriety or impropriety of ever attempting to preserve an exposed pulp, is to be determined by a careful consideration of two questions: 1. Is there any substance, which can be permanently retained in the tooth cavity, which the pulp will tolerate as a substitute for its wall of dentine? 2. Is the pulp (the lining membrane being included) capable of depositing dentine, or other bony tissue, for its own protection? Each of these questions must be answered by observation and experience. Theory can, and does give much light on the subject; but it must be verified by experience, before we can rely implicitly on it.

In answer to the first question, it may be stated that there are well authenticated cases, in which the pulps have retained their vitality for months, and even for years, though in contact with a foreign body introduced in filling the teeth. But even if this question is answered in the negative, let us examine the second. All, I believe, admit that the pulp is the organ which deposits the bony matter of the tooth. Now, when the pulp is exposed, does it lose all ability to perform its functions? It yet retains its vitality and is often found perfectly healthy. Or is it merely the point exposed that has lost its function? Why should it lose it, while it is still alive and healthy? True, it is no longer connected with the dentine; but we have seen that the periosteum will deposit bone when transplanted to soft tissue. The membrane at the point of exposure still retains its vital connection with the general circulation. It receives ossific matter, just as it did before exposure. Is there any physiological reason why it should not deposit it, just as the periosteum does, if it be thoroughly protected from external or foreign irritating agents? Or, if the part exposed does lose its function by exposure, is there any reason why the parts around the border of the orifice can not throw out osseous matter, and thus close the orifice? The periosteum can unite fractures, without exact apposition of the fractured parts. Recollect that the question refers only to cases of slight exposure. And when the exposure is slight, and the pulp and membrane are healthy, and the decayed matter is all removed, and irritating agents are carefully excluded, wherein does the case differ from that of a compound fracture? The circulation of the pulp is the same as that of the soft parts around a fracture, with this advantage, that its vessels are entire, while some of those concerned in the other case are ruptured. No one is surprised when a compound fracture heals by osseous union, for the simple reason that it is a common occurrence. When a fracture occurs, the parts are adjusted, and the patient is nursed, that it may heal. This has been the practice, from time immemorial. But when the pulp is exposed, the tooth is extracted, or the pulp is destroyed, or left to die. From time immemorial, no one has cared for it. Hence comparatively few cases of recovery by osseous protection have been observed.

This brings us exactly to the turning point of the whole question. Have any cases been observed in which the pulp has protected itself, after exposure, by a deposit of dentine, or other bony tissue? And here we will be allowed to refer to names. And, in doing so, we only aim at the attainment of truth. And we have just as much respect for those with whom we differ in opinion, as for those with whom we agree. We will not attempt to give the views of many members of the profession; and as the discussions on the subject of exposed pulp, at the Saratoga meeting, are still fresh in the minds of our readers, we will notice, a little, the views expressed there.

Dr. Taft, the first speaker on the subject, in favorable cases, fills, temporarily, over exposed pulps, and claims that, in many cases, they are eventually covered by a bony deposit.

Dr. Atkinson sets up the same claim, and proposes to show cases of secondary dentine.

Dr. Dwinelle "alluded to specimens in his possession, where large portions of secondary dentine had been deposited." He also "gave a case where the nerve bled, was filled, and on being opened, a secondary formation of dentine was found." He states, farther, that he had "filled many cases, and examined them, and found secondary dentine." He "had examined such cases microscopically, and had specimens." He had "practiced filling such cases for years, with the greatest success."

Dr. Morgan "has often found secondary dentine in cases where a tooth has been filled over exposed pulps."

Many other members of the profession, that we might name, advocate the same practice, and claim like success. None of them maintain that it would do to treat all, or even a majority of exposed pulps, this way. But they do maintain that if some teeth can be thus saved, it is worth while to make the effort. And they hope that still farther light will be gained, so that it will be easier to distinguish those which can be saved from those which can not.

On the other hand, we are told that Dr. Westcott has "met with no success," in this treatment for exposed pulp. How extensively and carefully he has tried it, we are not told; but, in the absence of information, we are to presume that he has given it a fair investigation.

Dr. Wetherbee "doubted the practicability of saving exposed nerves." He has "never seen a case of secondary dentine formed to protect a nerve."

"Dr. Rogers had never known of a case, where the pulp was wounded, to be restored by the use of caps, or any other method." He "thinks nine tenths of the pulps treated with a view to save their vitality, even by the best operators, fail."

Others, on both sides of the question, might be referred to, but these must suffice, as this article is already too long.

To the candid reader, a few questions would naturally present themselves at this point. Are those who advocate the preservative treatment of exposed pulps competent to judge accurately, in regard to the success or failure of their treatment? If they are not, is it because their ability to observe is defective, or because the subject is a difficult one? It can not be the latter; for it is easy to see an exposed pulp in some cavities; and, when it is wounded, the hemorrhage clearly reveals the fact of exposure. Nor is it difficult, after removing a temporary filling, to ascertain whether or not the pulp cavity is closed. New deposits of dentine, if any exist, are easily recognized. And if the pulp is dead, the odor, as referred to by Dr. Rogers, will generally reveal the fact.

We conclude, then, that those who advocate the preservative treatment, are as competent to observe facts, as those who recommend extirpation. They can see as well; and their sense of smell is just as acute. They can probably detect a failure as readily as their brethren; and they have just as little interest in practicing erroneously. They are not at all superior to their brethren, however. But let us look at the testimony given. One party testifies, individually, that they think it reasonable that the pulp, in favorable cases, should be protected by a new deposition of bony tissue, and that they have seen many cases in which it has been thus protected. The others tell us that they think it unreasonable, and that they have never seen a case in which it has occurred. Shall our verdict be in accordance with that of his Honor the Mayor, who fined Patrick, because two witnesses said they saw him strike Tim., while a dozen said they didn't see him do it?

